# Nutritional, physicochemical, and functional properties of protein concentrate and isolate of newly‐developed Bambara groundnut (*Vigna subterrenea* L.) cultivars

**DOI:** 10.1002/fsn3.552

**Published:** 2017-11-24

**Authors:** Olaposi R. Adeleke, Oladipupo Q. Adiamo, Olumide S. Fawale

**Affiliations:** ^1^ Department of Food Science and Technology Faculty of Technology Obafemi Awolowo University Ile‐Ife Osun State Nigeria; ^2^ Nigerian Institute for Oceanography and Marine Research Lagos Nigeria

**Keywords:** bambara groundnut cultivars, Defatted flour, Functional properties, Nutritional properties, Physicochemical properties, Protein concentrate

## Abstract

Bambara groundnut is an indigenous African vegetable grown mainly for human food and animal feed due to its high protein content. Different factors like varieties and origin can influence the chemical composition of Bambara groundnut cultivars. Therefore, the aims of this study are to produce defatted flour and protein concentrate from newly developed Bambara groundnut cultivars [Accessions No: TVSU 5 – Bambara Groundnut White (BGW) and TVSU 146 – Bambara Groundnut Brown (BGB)] and compare their nutritional, physicochemical, and functional properties with market sample [Bambara groundnut commercial (BGC)]. Higher protein content was observed in BGW (20.73%) and BGB (20.14%) as compared to BGC (18.50%). Also, the fat and ash contents of BGB and BGW were higher than that of BGC. Also, the new varieties were found to contain higher levels of some essential fatty acids such as linoleic and linolenic acids. The concentration of thiamine, riboflavin, niacin, pantothenic, ascorbic acids, pyrodoxine, alpha tocopherol, and vitamin K were also significantly higher in the two new varieties. The new varieties were good sources of magnesium, calcium, iron, manganese, sodium, and potassium. The oil and water absorption and swelling capacities of whole, defatted, and protein concentrate flour of the new varieties increase with increase in temperature. The defatted flour and protein concentrate of brown Bambara groundnut was found to exhibit high emulsifying activity and stability at different pH's and salt concentrations. The new varieties possess significantly higher foaming capacity and stability than the commercial variety. The results obtained from this study have shown the potential for the industrial and household use of the new Bambara groundnut cultivars into shelf stable protein products and could be a useful ingredient in food formulations.

## 1 INTRODUCTION

In human diet, legumes are being ranked next to cereals as sources of calorie and protein. With an exponential increase in population growth, decline per head capital availability and bad weather conditions, the production of common legumes like cowpeas might be inadequate (Atiku & Mohammed, 2004). The need therefore arises for the development and utilization of underutilized legume seeds which are abundant in Nigeria and are under‐exploited. Bambara groundnut (*Vigna subterrenea, L*.) falls into this group of underutilized species of plants.

Bambara groundnut (*Vigna subterrenea, L*.) is an indigenous African vegetable cultivated principally by farmers as a “famine food.” The high tolerance for drought and poor soil enables Bambara to grow under conditions unsuitable for groundnut (Agbenorhevi, Oduro, Ellis, Aodakpi, & Eleblu, 2007). In Nigeria, Bambara groundnut has found various food uses: it can be fried or boiled and eaten as snack or pounded into flour and used in the preparation of soup, porridge, and various fried or steamed food products such as “akara, moin‐moin” and “okpa.” It has also been used in the preparation of local food drink such as “kunnu” and “tuwo.” Reports has it that Bambara groundnut flour has been used in making bread in Zambia (Adebowale, Adeyemi, & Oshodi, 2005) and it was also observed that milk prepared from Bambara groundnut gave a preferred flavor when compared with milk from soybean, cowpea, and pigeon pea (Piyarat, 2008). The animal feed and haulm potentials of Bambara groundnut has long been discovered (Adebowale & Lawal, 2004) and its suitability for animal grazing which is basically due to the abundance of nitrogen and phosphorus in its leaves has also been reported (Bamishaiye, Adegbola, & Bamishaiye, 2011). Atiku and Mohammed (2004) noted that in the North Eastern Nigeria, Bambara groundnut is not only consumed as food but also used for medicinal purposes. Research efforts in Nigeria have only focused on the agronomy and little or no attention has been paid to the chemical composition and functional properties of protein products of some newly developed varieties of Bambara groundnut in spite of its growing importance.

However, available data on the chemical composition and nutritional properties are limited to the commonly used variety (commercial sample) and these parameters may vary among same sample of different varieties. The changes in the properties may be influenced by the variety/genetic origin of the varieties as well as climatic conditions, soil, pesticides, and fertilizers employed in the production of the crop. Therefore, the objective of the present research was to study the chemical compositions and nutritional properties of two newly developed Bambara groundnut cultivars and evaluate the functional properties of their defatted flour and protein concentrate. This study could provide some basic information, which would help determine an application for the newly developed cultivars as source of plant protein in food products.

## 2 MATERIALS AND METHODS

### 2.1 Collection of raw materials

The two new varieties of Bambara groundnut seeds (Accessions No: TVSU 5 – Bambara Groundnut White (BGW) and TVSU 146 – Bambara Groundnut Brown (BGB) used for this study were collected from the International Institute of Tropical Agriculture (IITA), Ibadan, Oyo State, Nigeria. The seeds were planted on a farmland at Kelebe, Osogbo, Osun State, Nigeria and the ripe, matured dried pods were harvested after 160 days. The seeds were dehulled from the sundried pods while the Bambara groundnut commercial (BGC) seeds were purchased from a local market in Oyo, Nigeria. Adhering dirt were removed from the seeds by washing with clean tap water, sun dried and stored in airtight containers for analysis. All chemicals used were of analytical grade and obtained from Sigma chemicals, (St. Louis, MO, U.S.A.).

### 2.2 Sample preparation

#### 2.2.1 Preparation of defatted flour

Bambara groundnut defatted flour was prepared using a modified method of Sathe (1994). The cleaned Bambara groundnut were ground in a Warring blender (BLG‐450, Binatone, Shenzhen, China) and the flour was defatted with cold (4°C) acetone (flour to solvent ratio 1:5 w/v) with constant magnetic stirring provided for 4 hr. The trace of residual acetone was removed by placing the defatted flour inside a fume cupboard for 6 hr to dry. A fine powder (moisture content: 10.92%) was obtained by grounding the defatted flakes, sieved through a size mesh of 150 μm, packed in plastic tubes and stored at −10°C.

#### 2.2.2 Preparation of protein concentrate

Bambara groundnut concentrate samples were prepared by a modification of the method described by Cheftel, Lug, and Lorient (1985). A ratio of 1:10 flour to water was stirred on a magnetic stirrer for 10 min, and the resultant slurry was adjusted to pH 4.0 and centrifuged (MSE, Harrier 15/80, Sydenham, London, U.K.) at 3,500*g* for 30 min. The precipitate pH was adjusted to 7.0, was washed twice with distilled water, centrifuged at 3,500*g* for 10 min and dried in an oven at 45°C for 8 hr to obtain the protein concentrate.

### 2.3 Proximate composition

Crude protein, moisture, fat, and ash contents of whole and defatted flour of BGB, BGW, and BGC were determined according to AOAC (2000).

### 2.4 Physicochemical and functional properties

Bulk density (BD) was determined by the method described by Okezie and Bello (1988). A method described by Adepeju, Gbadamosi, Adeniran, and Omobuwajo (2011) was used in determining the water absorption capacity (WAC) at room temperature (25°C) and varying temperature (60–90°C). Two gram (2 g) sample was mixed with 10 ml distilled water, centrifuged at 4,000*g* for 30 min and the water absorption was expressed as percentage increase in the sample weight. Oil absorption capacity (OAC) was determined by the centrifugal method described by Beuchat (1977). The method of Sathe and Salunkhe (1981) was employed for the determination of gelling concentration. The swelling capacity (SC) was determined using the method described by Takashi and Sieb (1988) with slight modification. Exactly, 1 g of sample was mixed with 10 ml distilled water and the slurry was heated at a constant temperature (60, 70, 80, and 90°C) in a water bath for 15 min, centrifuged at 3,000*g* for 10 min and the SC was expressed as percentage increase in sample weight.

### 2.5 Nutritional properties

#### 2.5.1 Vitamins determination

##### Standard preparation

A stock solution of 1.0 mg/ml of each water soluble vitamin standards **(**thiamine (Vitamin B_1_), riboflavin (Vitamin B_2_), nicotinamide (Vitamin B_3_), pantothenic acid (Vitamin B_5_), pyridoxine (Vitamin B_6_), cyanocobalamin (Vitamin B_12_), and ascorbic acid (vitamin C)) was prepared by weighing 10 mg of each vitamin powder and 10 ml of deionized water was added. The concentration of stock solution of vitamin B_2_ was decreased to 0.25 mg/ml in deionized water while vitamin B_9_ was prepared using 0.2 mol/L of KHCO_3_ instead of deionized water to make a solution of 0.5 mg/ml due to their limited solubility in water. A stock solution of 1.0 mg/ml for each fat‐soluble vitamin standards of vitamins A, D, and E was prepared by weighing 10 mg of each standard vitamin and 10 ml of CH_3_OH was added. Acetone and CH_2_Cl_2_ was used in preparing the standard for vitamin K_1_ instead of CH_3_OH. The stock standards of fat soluble vitamins were stored in the dark. A mixture of CH_**3**_OH:CH_2_Cl_2_ (1:1, v/v) was used for preparation of working standard for fat‐soluble vitamins while the water‐soluble vitamin working standards were prepared from the stock standards on the day of use by diluting with deionized water.

##### Sample preparation

The water‐soluble vitamins were extracted by weighing exactly 0.100 g sample into 100 ml volumetric flasks; then 80 ml of water was added. After 15 min of extraction, water was added to the mark and the solution was filtered through a 0.2 μm filter prior to injection into the HPLC. About 0.125 g of Bambara groundnut flours were weighed separately into 10 ml volumetric flasks followed by addition of 8 ml of CH_3_OH:CH_2_Cl_2_ (1:1, v/v) to each flask to extract the fat soluble vitamins. After 15 min of extraction, CH_3_OH:CH_2_Cl_2_ (1:1, v/v) was added to the mark. The solution was filtered through 0.2 μm filter and the sample solutions were stored in a dark cupboard prior to injection into the HPLC.

##### HPLC conditions

For determination of water‐soluble vitamins, the conditions were; Column: Acclaim PA2, 5 μm, 120 Å, 4.6 × 150 mm (P/N 063197); Temperature: 25°C Mobile phase: A: CH_3_CN B: 0.25 mol/L phosphate buffer (about 3.4 g KH_2_PO_4_ was dissolved in 1 L of water, and pH was adjusted to 3.2 with H_3_PO_4_); Flow Rate: 1.0 ml/min; Injection Vol: 20 μl; UV Detection: Absorbance at 210, 245, 265, and 280 nm. Also the conditions for determination of fat‐soluble vitamins include; Column: Acclaim C18, 5 μm, 120 Å, 4.6 × 150 mm (P/N 059133); Temperature: 25°C; Mobile phase: A: CH_3_OH:CH_3_CN (8:2, v/v); B: MTBE; Flow Rate: 1.0 ml/min; Injection Vol: 20 μl; UV Detection: Wavelength‐switching absorbance at 265, 325, and 450 nm.

#### 2.5.2 Minerals content analysis

The main and trace elements of sample was determined using the method described in the AOAC (2000). Exactly 0.5 g of sample was weighed into a digestion tube and 10 ml of nitric/perchloric acid was added prior to mineral determination. The samples were digested at 150°C until a clear fume was obtained and washed into a standard 50 ml volumetric flask and distilled water was added up to the mark. Atomic absorption spectrophotometer (210VGP, Buck Scientific incorporation, Norwalk, Connecticut, USA) was used in the determination of calcium (Ca), magnesium (Mg), copper (Cu), zinc (Zn), manganese (Mn), cadmium (Cd), chromium (Cr), and iron (Fe) was determined using a spectrophotometer (Spectro 20D plus RS‐232C, Labomed incorporation, Culver city, CA, USA) and sodium (Na) and potassium (K) were determined using a flame photometer (2655‐00, Coring Inc., USA.).

#### 2.5.3 Fatty acid determination

The fatty acid composition of the samples was determined using Gas Chromatography (GC) of the methyl ester method (Sun, Han, Yan, Yang, & Tetsuo, 2008). Exactly, 0.5 g of each sample was weighed, mixed with 1.5 ml hexane overnight and the mixture was centrifuged at 7,000 rpm for 5 min. About 350 lL of sodium methoxide solution was added to the supernatant, vortexed thoroughly and mixed for 1 hr. The supernatant obtained after centrifuging at 7,000 rpm for 5 min was filtered into the special sample bottle for GC detectors. HP INNOWax Column (30 mm × 0.25 mm × 0.25 mm) was used for the GC analysis with nitrogen, hydrogen, and air as the carrier gases for 20 min and the injection volume was 1 lL. The percentage of fatty acid components was calculated using the area normalization method.

### 2.6 Effect of pH and NaCl concentration on emulsifying activity index and emulsion stability index of defatted and protein concentrate of Bambara groundnut cultivars

The effect of pH and salt concentration on emulsifying activity index (EAI) was determined by the method described by Gbadamosi, Abiose, and Aluko (2012) with some modifications. At different NaCl concentrations (0.0, 0.5, and 1.0 mol/L), approximately 500 mg of the samples was dispersed in 100 ml of distilled water. The pH of the protein solution was then adjusted with either 1 N HCl or 1 N NaOH to pH 2, 4, 6, 8, and 10 separately. The protein solution was homogenized using a blender (SN2200 Qlink, Beijing, China) set at high speed for 60 s after been mixed with 50 ml of pure Gino oil. After homogenization, 50 µl of the aliquot of the emulsion was transferred from the bottom of the blender and mixed with 5 ml of 0.1% sodium dodecyl sulfate (SDS) solution. The absorbance of the resulting solution was measured at 500 nm using spectrophotometer (722‐2000 Spectronic 20D, Jiangsu, China). The EAI was calculated from the absorbance obtained as shown in the formula below:

$$\text{Emulsifying activity index}\;\left(m^{2}/g\right)=\frac{2\times 2.303\times A}{0.25\times \text{sample weight (g)}}$$

To determine the emulsion stability, the emulsions was allowed to stand for 10 min at room temperature and the ESI was determined as described above and it was expressed based on the absorbance at 0, 10 min and the time difference as shown in the formula below:

$$\text{Emulsion stability index}\left(\%\right)=\frac{AA\times \Delta t}{A-AA}$$

where *A* is the absorbance at 0 min after homogenization; AA is the absorbance at 10 min after homogenization; Δ*t* = 10 min; and Δ*A* = A–AA.

### 2.7 Effect of pH and NaCl concentration on foam capacity and stability of defatted and protein concentrate of Bambara groundnut cultivars

The effect of pH and salt concentration on foam capacity (FC) and foam stability (FS) was determined by a modification of the method described by Chavan, McKenzie, and Shahidi (2001). At different NaCl concentrations (0.0, 0.5, and 1.0 mol/L), approximately 500 mg of the samples was dispersed in 100 ml of distilled water. The pH of the protein solution was then adjusted with either 1 N HCl or 1 N NaOH to pH 2, 4, 6, 8, and 10 separately. The protein solution was homogenized using a blender (SN2200 Qlink, Beijing, China) set at high speed for 2 min and then poured into 250 ml measuring cylinder. The percentage ratio of the volume increase to that of the original volume of protein solution in the measuring cylinder was calculated and expressed as FC while the FS was expressed as percentage of the volume of foam remaining in the measuring cylinder to that of the original volume after 30 min of quiescent period.

$$\begin{array}{cc} & \text{Foaming capacity}\left(\%\right)\\ & =\frac{\left(\text{volume after whipping}-\text{volume before whipping}\right)\text{ml}}{\left(\text{volume before whipping}\right)\text{ml}}\times 100 \end{array}$$

$$\begin{array}{cc} & \text{Foaming stability}\left(\%\right)\\ & =\frac{\left(\text{volume after whipping}-\text{volume after standing}\right)\text{ml}}{(\text{volume after whipping}-\text{volume before whipping})\text{ml}}\times 100 \end{array}$$

### 2.8 Statistical analysis

Statistical analysis of data collected in triplicate was carried out using Data collected analysis of variance (ANOVA) and the differences between the treatment means were separated using Duncan's multiple range tests at a level considered to be significant at *p* < .05.

## 3 RESULTS AND DISCUSSION

### 3.1 Chemical composition

The chemical composition of the Bambara groundnut cultivars is shown in Table 1. The protein content of the samples ranged between 18.50% and 20.73%, with the commercial sample (BGC) exhibiting the lowest (18.50%) and BGW the highest (20.73%). The protein contents of BGW (20.73%) and BGB (20.14%) were significantly (*p* < .05) higher than that of the commercial sample (BGC: 18.50%). The values obtained in this study agree with what was reported for commercial Bambara groundnut seeds by Abdulsalami and Sherriff (2010). According to the FAO/WHO (2007), adequate dietary protein is essential during growth when new tissue proteins are being synthesized. The two new varieties owing to their high contents of protein could be utilized in the formulation of protein rich diet or for the supplementation of diets low in protein contents. The fat contents of the samples ranged between 6.01% and 7.93% with BGB exhibiting significantly (*p* < .05) highest value. These values obtained were comparable with that obtained for Bambara groundnut seeds (Eltayeb, Ali, Abou‐Arab, & Abu‐Salem, 2011). Essential fatty acids and energy which serves as a vehicle for fat‐soluble vitamins and facilitates their absorption are been produced by dietary fat. The ash composition of sample BGW (5.15%) was significantly (*p* < .05) higher than that of BGB (4.33%) and BGC (4.18%) and these values were within the range (3.0%–4.5%) reported by Fadahunsi, Jonathan, and Garuba (2010) for Bambara groundnut. Ash is an indication of the mineral contents of the samples and the high values obtained in these new varieties indicates that it may serve as sources of micro and macro elements. Commercial sample (BGC) had the highest carbohydrate content and energy values as compared to that of BGB and BGW (*p* < .05). The values obtained for BGW and BGB were still higher than 46.5% reported by Okonkwo and Opara (2011) for Bambara groundnut.

**Table 1:** Nutritional and chemical composition^a^ of commercial and newly developed Bambara groundnut cultivars

Sample	Moisture (%)	Protein (%)	Fat (%)	Ash (%)	Crude fiber (%)	Carbohydrate (%)	Energy value (kcal)	Dry matter (g/100 g)
BGC	7.19 ± 0.50^a^	18.50 ± 0.26^a^	6.01 ± 0.34^a^	4.18 ± 0.10^a^	3.64 ± 0.27^c^	60.48 ± 0.50^c^	6.08 ± 0.11^c^	92.81 ± 0.43^c^
BGW	10.10 ± 0.17^b^	20.73 ± 0.57^c^	7.93 ± 0.64^b^	5.15 ± 0.64^c^	2.11 ± 0.11^a^	53.98 ± 1.13^b^	5.43 ± 0.09^b^	89.90 ± 0.17^b^
BGB	11.87 ± 0.85^c^	20.14 ± 0.88^b^	8.73 ± 1.17^c^	4.33 ± 0.76^b^	2.20 ± 0.10^b^	52.73 ± 1.58^a^	4.12 ± 0.01^a^	88.13 ± 0.32^a^

Sample	Vitamin B_1_	Vitamin B_2_	Vitamin B_3_	Vitamin B_5_	Vitamin B_6_	Vitamin B_9_	Vitamin C	Vitamin E	Vitamin K
BGC	0.56 ± 0.09^a^	0.10 ± 0.00^a^	11.40 ± 0.34^a^	1.25 ± 0.05^a^	0.35 ± 0.01a	0.21 ± 0.08^a^	0.22 ± 0.04^a^	8.33 ± 0.33^c^	0.00 ± 0.00^a^
BGW	0.64 ± 0.08^bc^	0.12 ± 0.00^b^	13.05 ± 0.45^c^	1.84 ± 0.08^bc^	0.43 ± 0.05^c^	0.25 ± 0.01^b^	0.27 ± 0.08^b^	3.31 ± 0.12^a^	0.001 ± 0.00^a^
BGB	0.61 ± 0.01^b^	0.13 ± 0.09^c^	12.74 ± 0.21^b^	1.80 ± 0.04^b^	0.44 ± 0.08^bc^	0.25 ± 0.01^b^	0.27 ± 0.01^b^	3.42 ± 0.67^b^	0.001 ± 0.00^a^

Sample	Magnesium	Calcium	Sodium	Copper	Iron	Zinc	Manganese	Potassium	Cadmium	Chromium
BGC	7.02 ± 0.10^a^	0.23 ± 0.50^a^	2.18 ± 0.14^a^	0.44 ± 0.05^c^	2.51 ± 0.02^a^	0.81 ± 0.50^c^	0.06 ± 0.01^a^	92.43 ± 1.14^a^	2.02 ± 0.50^a^	1.98 ± 0.00^a^
BGW	8.56 ± 4.50^c^	1.40 ± 0.10^b^	2.30 ± 0.00^b^	0.13 ± 0.00^b^	6.67 ± 0.29^c^	0.23 ± 0.00^b^	0.18 ± 0.00^b^	147.00 ± 2.50^b^	2.00 ± 0.00^a^	2.00 ± 0.01^ab^
BGB	7.58 ± 0.10^b^	1.60 ± 0.00^c^	3.60 ± 0.00^c^	0.09 ± 0.00^a^	5.52 ± 0.01^b^	0.27 ± 0.00^a^	0.26 ± 0.00^c^	183.00 ± 1.50^c^	2.50 ± 0.00^b^	2.00 ± 0.00^ab^

Sample	Myristic	Palmitic	Behenic	Stearic	SFA (%)	Oleic	Linoleic	Linolenic	Palmitoleic	UFA (%)
BGC	0.27 ± 0.04^c^	9.97 ± 0.69^b^	5.41 ± 0.47^c^	4.14 ± 0.22^c^	19.55	54.17 ± 0.77^c^	21.14 ± 0.99^a^	0.82 ± 0.01^b^	3.41 ± 047^c^	79.86
BGW	0.21 ± 0.04^b^	10.37 ± 0.14^c^	3.00 ± 0.49^a^	4.10 ± 0.42^b^	17.63	52.02 ± 0.42^b^	25.95 ± 0.70^c^	0.30 ± 0.01^a^	0.29 ± 0.18^a^	82.33
BGB	0.14 ± 0.02^a^	9.72 ± 0.22^a^	4.41 ± 0.23^b^	3.72 ± 0.33^a^	10.99	51.98 ± 0.20^a^	25.10 ± 0.40^b^	0.87 ± 0.20^c^	0.65 ± 0.12^b^	83.01

a Values are means ± standard deviation of three determinations. Means followed by the same letter within the same rows are not significantly (*p* < .05) different according to LSD test. White bambara groundnut sample (BGW); Brown bambara groundnut sample (BGB); Control bambara groundnut sample (BGC).

### 3.2 Nutritional composition

#### 3.2.1 Vitamins composition

The vitamin contents of samples BGW, BGB, and BGC are shown in Table 1. The result of the B‐vitamins showed that BGW and BGB had significantly (*p* < .05) higher B‐vitamin contents as compared to the commercial sample, BGC. Vitamin B_3_ was highest among all the vitamins in the three samples studied with BGW exhibiting highest value of 13.05 as compared to that BGB (12.74) and BGC (11.40). The results of this investigation were significantly (*p* < .05) higher than the value of 0.88 mg/100 g reported for vitamin B_3_ in Bambara groundnut seed by Odeghe, Adumanya, Obi‐Adumanya, and Chukwu (2012). Varietal differences as well as determination procedure could be the reason for the difference. According to FAO/WHO (2004), the recommended daily allowance for vitamin B_3_ (water soluble vitamin) that occurs in the form of niacin, nicotinic acid, and nicotinamide in adult males and female is 14–16 mg niacin equivalent. Therefore, the high vitamin B_3_ content found in the two new varieties implies they could be a better source of niacin compared to the commercial sample. Also, the vitamin C of BGC (0.22) were significantly (*p* < .05) increased to 0.27 in BGW and BGB. However, a significantly (*p* < .05) lower value was observed in the vitamin E content of BGW (3.31) and BGB (3.42) as compared to BGC (8.33). Dietary deficiency of vitamin E is not normally encountered (FAO/WHO, 2004). The requirement of vitamin E suggested was 8–10 mg tocopherol per day depending on the edible oil used. Therefore, the values obtained in these new varieties were lower than the recommended daily allowance of vitamin E.

#### 3.2.2 Minerals composition

The results of the mineral composition showed that the newly developed Bambara groundnut cultivars have significantly higher (*p* < .05) macro mineral (magnesium, calcium, sodium, and potassium) contents compared to the commercial sample (Table 1). The amount of potassium was the highest among the minerals in all the three samples with BGB having significantly (*p* < .05) highest value of 183 ppm. The results obtained were higher than 92.43 ppm reported for Bambara groundnut seeds by Abdulsalami and Sherriff (2010) and 160.00 ppm reported by Bamishaiye et al. (2011). Potassium is an essential mineral, widespread in nature and the recommended daily allowance (RDA) in adult is 3.5 g/day (Bender, 2006). Therefore, the potassium contents obtained in the three samples were lower than the RDA in adult. However, the lowest among the macro minerals is calcium with BGC, BGW, and BGB having values of 0.23, 1.40, and 1.60, respectively, (*p* < .05). The results obtained for the two new varieties were still higher than 0.23 ppm and 0.79 ppm reported by Abdulsalami and Sherriff (2010) and Odeghe et al. (2012), respectively, for raw Bambara groundnut cultivars.

Among the micro minerals, iron content was the in the three samples and this ranged between 2.51 and 6.67 ppm, with BGW having significantly (*p* < .05) highest value. The body requires about 1.5–2.2 mg/day of iron (Belitz, Grosch, & Schierberle, 2010). According to Abdulsalami and Sherriff (2010), raw and processed Bambara groundnut cultivars contained 5.5 and 2.96 ppm of iron, respectively. Hemoglobin and myoglobin pigments as well as some enzymes which are the major constituents of iron must be taken at about 15 mg/day by an average human being (Rao, 2007). The lowest micro mineral was copper with commercial sample, BGC (0.44 ppm) exhibiting significantly (*p* < .05) higher value compared to BGW (0.13 ppm) and BGB (0.09 ppm). The results obtained for samples BGW and BGB were lower than that of Bambara groundnut seed (0.41 ppm) reported by Abdulsalami and Sherriff (2010). In the formation of oxido‐reductase enzyme, copper has been seen to be a vital element as it helps in the catalysis of iron II to iron III. This reaction has been found to be very important because it is only in this form that protein can be transported to the liver (Bender, 2006). According to Chiplonkar, Agte, and Mengale (2003), the recommended dietary allowance (RDA) of copper is about 2 mg/day, but in all the samples copper contents was found to be lower than the RDA level.

#### 3.2.3 Fatty acids profile

The fatty acids profile of samples BGC, BGB, and BGW is shown in Table 1. Palmitic, oleic, and linoleic acids were found to be the most abundant in the samples. The monounsaturated fatty acid (oleic) was the most abundant fatty acid in Bambara groundnut constituting more than half of the total fatty acids. The commercial sample showed the highest level of oleic acid when compared to the two new varieties and the differences among the samples with respect to oleic acid were significant (*p* < .05). The levels of linolenic, palmitoleic and myristic in samples BGC, BGW, and BGB were 0.82, 3.41 and 0.27%, 0.30, 0.29 and 0.21% and 0.87, 0.65 and 0.14%, respectively. The results obtained were in conformity with the report by Okonkwo and Opara (2011) and Minka and Bruneteau (2000) that linoleic, palmitic, and linolenic were predominant types of fatty acids present in Bambara groundnut seeds. It was also observed that the saturated fatty acids constitute about 20% of the total fatty acids while unsaturated fatty acids make up about 80%. For generation of cellular energy and biosynthesis of membrane lipids and lipid mediators in the body, fatty acids are used (Ratnayake & Galli, 2009). Two of the essential fatty acids (Linoleic and oleic acids) were found to be present in abundant quantities in the new Bambara groundnut seeds while the stability of the Bambara groundnut oil might be adversely affected by the higher proportion of unsaturated fatty acids present. Oxidation of the unsaturated fatty acids at the double bonds makes this oil to be prone to the development of rancid flavor. The suitability of such oil for light cooking might be due to the awareness of the consumption of diet containing higher ratio of polyunsaturated fatty acids to saturated fatty acids which are now being advocated due to the high incidence of coronary diseases such as atherosclerosis.

### 3.3 Functional properties

#### 3.3.1 Bulk density (BD) and pH

The BD ranged between 0.58 and 0.73 g/ml; 0.77–0.82 g/ml and 0.62–0.70 g/ml for whole, defatted and concentrates flour, respectively, (Table 2). The defatted sample (BGB) exhibited the highest BD (0.82 g/ml) while the whole flour (BGC) had the lowest bulk density (0.58 g/ml). The values obtained were comparable to 0.56 and 0.62 g/ml reported by Eltayeb et al. (2011) for Bambara groundnut flour but higher than that of conophor flour (0.41 g/ml) and kariya protein concentrates (0.57 g/ml) as reported by Gbadamosi (2008) and Adiamo, Gbadamosi, and Abiose (2016a), respectively. The presence of higher proportion of carbohydrate in defatted flour may be responsible for the high BD demonstrated by defatted Bambara flour samples. Starch polymer structure has been seen to influence BD and loose starch polymer could result in low bulk density (Plaanmi, 1997). The increase observed in the rate of dispersion has a result of high bulk density is important in the reconstitution of flour in hot water to produce dough (Akinjayeju & Enude, 2002). The aqueous solutions of whole and defatted flour of Bambara groundnut seeds (BGC, BGW, and BGB) were acidic as shown in Table 2. The pH values range between 6.30 and 6.67. The pH values of the aqueous solutions of the protein concentrate (BGC, BGW, and BGB) ranged between 7.12 and 7.25. The adjustment of pH to 7 during the preparation of concentrate may influence its pH values in aqueous solution. Adepeju et al. (2011) reported that some functional properties such as solubility, emulsifying activity and foaming properties are affected by pH.

**Table 2:** Functional properties^a^ of whole, defatted, and protein concentrate of commercial and newly developed Bambara groundnut flour

Functional Properties	Whole			Defatted			Concentrate		
	BGC	BGW	BGB	BGC	BGW	BGB	BGC	BGW	BGB
BD (g/ml)	0.58 ± 0.02^a^	0.64 ± 0.04^b^	0.73 ± 0.01^c^	0.77 ± 0.00^a^	0.79 ± 0.00^b^	0.82 ± 0.32^c^	0.62 ± 0.01^a^	0.63 ± 0.01^ab^	0.70 ± 0.04^c^
OAC (%)	69.07 ± 0.09^b^	73.32 ± 0.43^c^	63.44 ± 0.06^a^	79.71 ± 0.07^b^	79.54 ± 0.56^b^	70.94 ± 0.17^a^	89.18 ± 0.21^a^	89.72 ± 0.03^ab^	103.66 ± 0.03^c^
pH	6.67 ± 0.05^c^	6.65 ± 0.15^b^	6.40 ± 0.00^a^	6.30 ± 0.00^a^	6.40 ± 0.00^b^	6.50 ± 0.00^c^	7.12 ± 0.05^a^	7.25 ± 0.0^bc^	7.20 ± 0.00^b^
WAC (%)	137.67 ± 0.15^b^	139.33 ± 0.08^c^	108.67 ± 0.05^a^	168.67 ± 0.00^c^	149.33 ± 0.05^a^	161.33 ± 0.05^b^	286.67 ± 0.05^b^	311.43 ± 0.25^c^	174.65 ± 0.05^a^
SC	2.52 ± 0.06^b^	2.71 ± 0.04^c^	2.39 ± 0.01^a^	9.72 ± 0.01^a^	10.14 ± 0.03^b^	10.94 ± 0.01^c^	2.64 ± 0.01^b^	3.19 ± 0.03^c^	1.96 ± 0.01^a^
LGC (%)	20.00 ± 0.00^b^	9.00 ± 0.00^a^	20.00 ± 0.00^b^	20.00 ± 0.00^a^	20.00 ± 0.00^a^	20.00 ± 0.00^a^	20.00 ± 0.00^c^	15.00 ± 0.00^a^	13.00 ± 0.00^b^
EAI (m^2^/g)	14.37 ± 0.23^b^	13.21 ± 0.44^a^	17.56 ± 0.28^c^	19.29 ± 0.11^a^	19.27 ± 0.34^a^	21.02 ± 0.21^b^	22.04 ± 0.28^c^	17.91 ± 0.10^a^	21.24 ± 0.24^b^
ESI (%)	127.14 ± 0.56^b^	82.16 ± 0.68^a^	164.800.77^c^	30.12 ± 0.02^b^	26.83 ± 0.68^a^	54.83 ± 0.50^c^	60.90 ± 0.38^a^	81.47 ± 0.30^c^	79.56 ± 0.55^b^
FC (%)	8.41 ± 0.11^a^	12.75 ± 0.05^c^	9.90 ± 0.57^b^	12.00 ± 0.034^a^	12.71 ± 0.06^a^	14.21 ± 0.06^b^	13.57 ± 0.03^a^	14.01 ± 0.01^b^	23.56 ± 0.09^c^
FS (%)	42.43 ± 0.10^a^	54.13 ± 0.47^c^	47.31 ± 0.50^b^	25.47 ± 0.42^a^	40.70 ± 0.55^b^	52.63 ± 0.06^c^	89.76 ± 0.03^a^	92.50 ± 0.01^b^	97.82 ± 0.05^c^

BD, Bulk Density; OAC, Oil Absorption Capacity; WAC, Water Absorption Capacity; SC, Swelling Capacity; LGC, Least Gelling Concentration; EAI, Emulsion Activity Index; ESI, Emulsifying Stability Index; FC, Foam Capacity; FS, Foam Stability.

a Values are means ± standard deviation of three determinations. Means followed by the same letter within the same rows are not significantly (*p* < .05) different according to LSD test. White bambara groundnut sample (BGW); Brown bambara groundnut sample (BGB); Control bambara groundnut sample (BGC).

#### 3.3.2 Oil absorption capacity (OAC)

The OAC of samples BGW and BGB were found to be 79.54 and 63.44%, respectively, while that of sample BGC was 69.07% (Table 2). The OAC of Bambara protein concentrate of BGB was the highest (103.66%). The OAC of defatted and protein concentrate of the three samples were higher than those of their raw samples. The OAC of Bambara groundnut flour products was higher than that of sandbox seed flour (65.50%–107.63%) as reported by Osungbade, Gbadamosi, and Adiamo (2016) but lower than that of jackfruit flour (230 and 350%) as reported for jackfruit flour by Odoemelam (2005). The low OAC of Bambara groundnut products flour might be due to low levels of hydrophobic proteins which show superior binding of lipids. The bridge caused by protein in fat and water emulsion may not make Bambara groundnut flour products a suitable ingredient in the cold meat industry particularly for sausages.

#### 3.3.3 Water absorption capacity (WAC)

The WAC ranged from 108.67 to 311.43% (Table 2). The concentrate of the three samples had the highest WAC which implies that increase in protein level increases the WAC of the products with sample BGW exhibiting the highest value of 311.43%. The higher WAC of Bambara groundnut concentrate flour may be due to the higher polar amino acid residues of protein having an affinity for water molecules (Yusuf, Ayedun, & Sanni, 2007). Protein concentrate was also observed by Gbadamosi (2008) to exhibit better water binding capacity to that of the raw conophor nut flour. Osundahunsi, Fagbemi, Kesselman, and Shimoni (2003) attributed this to the fact that protein concentrate had greater ability to swell, dissociate and unfold exposing additional binding sites, whereas the carbohydrate and other compounds of the protein flour may impair it. The incorporation of the new Bambara groundnut seed flours and its protein products into aqueous food formulations, especially those involving soup and dough handling might be due to its good WAC which is the ability of flour to absorb water and swell for improved consistency in food.

#### 3.3.4 Least gelation concentration (LGC)

The LGC in this study ranged from 9.0 to 20.0% with raw BGW having the lowest value (Table 2). Processing methods, the relative ratio of different constituents‐ protein, carbohydrate and lipids and the interaction between such components might be due to the variations observed in the LGC values and these may affect functional properties (Aremu, Olaofe, & Akintayo, 2006). (Aremu et al., 2006) reported that the LGC of some legume ranged between 13.0 and 16.0%. The LGC is the lowest protein concentration at which gel remained in inverted tube. Lower LGC results in better gelation ability of protein ingredient (Akintayo, Oshodi, & Esuruoso, 1999). The use of BGW flour for the formation of curd or as an additive to other gel forming materials in food products may be an asset owing to its low gelation concentration (Akintayo et al., 2002).

#### 3.3.5 Effect of temperature on water absorption and swelling capacity

**Figure 1:**
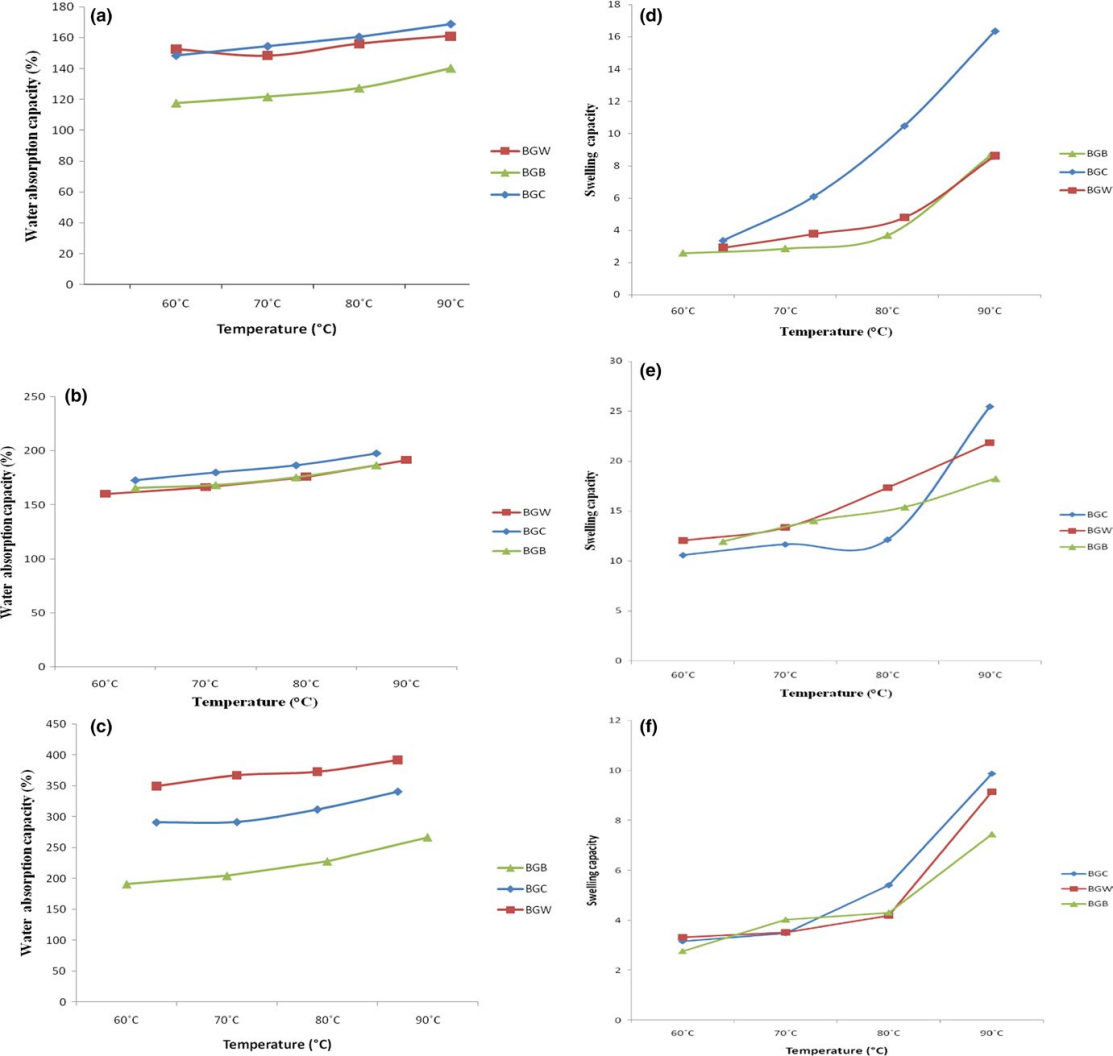
Influence of temperature on water absorption capacity (WAC) and swelling capacity (SC) of (a, d) whole, (b, e) defatted and (c, f) concentrate of Bambara groundnut cultivars, respectively

The effect of temperature on water absorption capacity of whole, defatted and protein concentrate of Bambara groundnut flour samples is shown in Figure 1. The water absorption capacities of whole, defatted, and concentrate flours increased with increase in temperature from 60 to 90°C. The samples of protein concentrate demonstrated the highest capacity to absorb water with sample BGW exhibiting the highest value (391.43%) at 90°C. The high WAC of both the defatted and the concentrate of Bambara groundnut flour suggest that it can be used as thickener in liquid and semi liquid foods because of their ability to absorb water for improved consistency in food (Fasasi, Adeyemi, & Fagbenro,^2007^; Osundahunsi et al., 2003).

The results showing the effect of different temperatures on the swelling capacity (SW) of Bambara groundnut samples are shown in Figure 1. As temperature increases, there is significant increase in the SW of whole, defatted, and protein concentrate in samples BGC, BGW, and BGB increased with increase in temperature from 60 to 90°C with defatted samples having the greatest values. The highest swelling capacity occurs in BGC concentrate (25.47) as compared to other samples at 90°C. The increase in temperature indicating differences in molecular organization within the granules results in an increase un swelling capacities of the flour (Agunbiade & Longe, 1999). Process conditions, nature of materials and type of treatment are the main conditions on which swelling capacities are dependent. The swelling capacities of samples increased with increase in temperature. Mune‐Mune, Minka, Mbome, and Etoa (2011) reported 0.95%–3.76% for Bambara groundnut flour while 91.17%–103.7% was reported for cowpea and yam‐bean (Agunbiade & Longe, 1999). The high swelling power achieved at 90 ºC suggests that water penetration into the starch granules and proteins can be achieved at elevated temperature. This could be useful in the manufacture of confectionery foods (that requires high swelling power).

### 3.4 Effect of pH and NaCl concentration on emulsifying activity and emulsifying stability index

**Figure 2:**
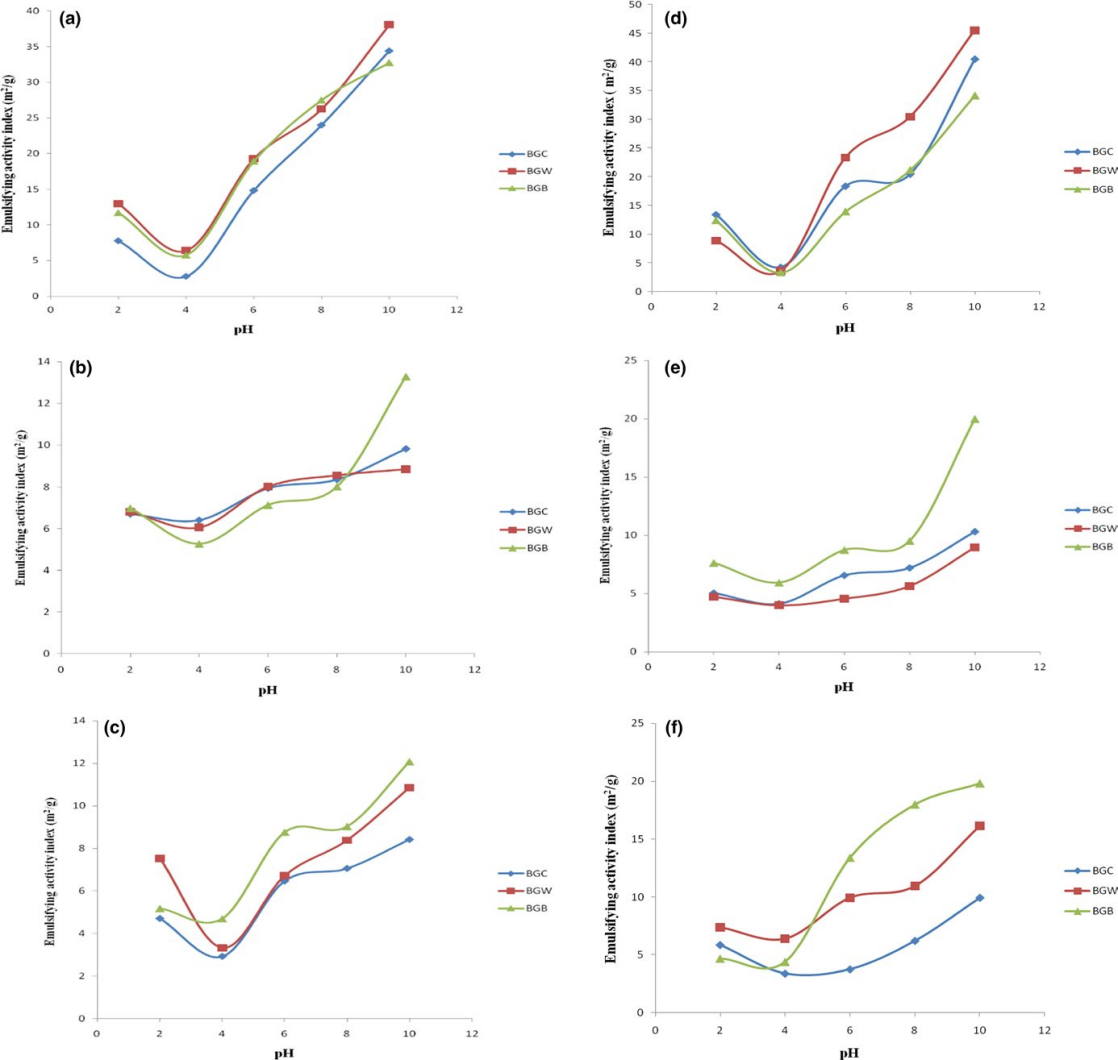
Emulsifying activity index (m^2^/g) of defatted and protein concentrate of Bambara groundnut cultivars at (a, d) 0.0 mol/L, (b, e) 0.5 mol/L, and (c, f) 1.0 mol/L NaCl concentration as a function of pH

**Figure 3:**
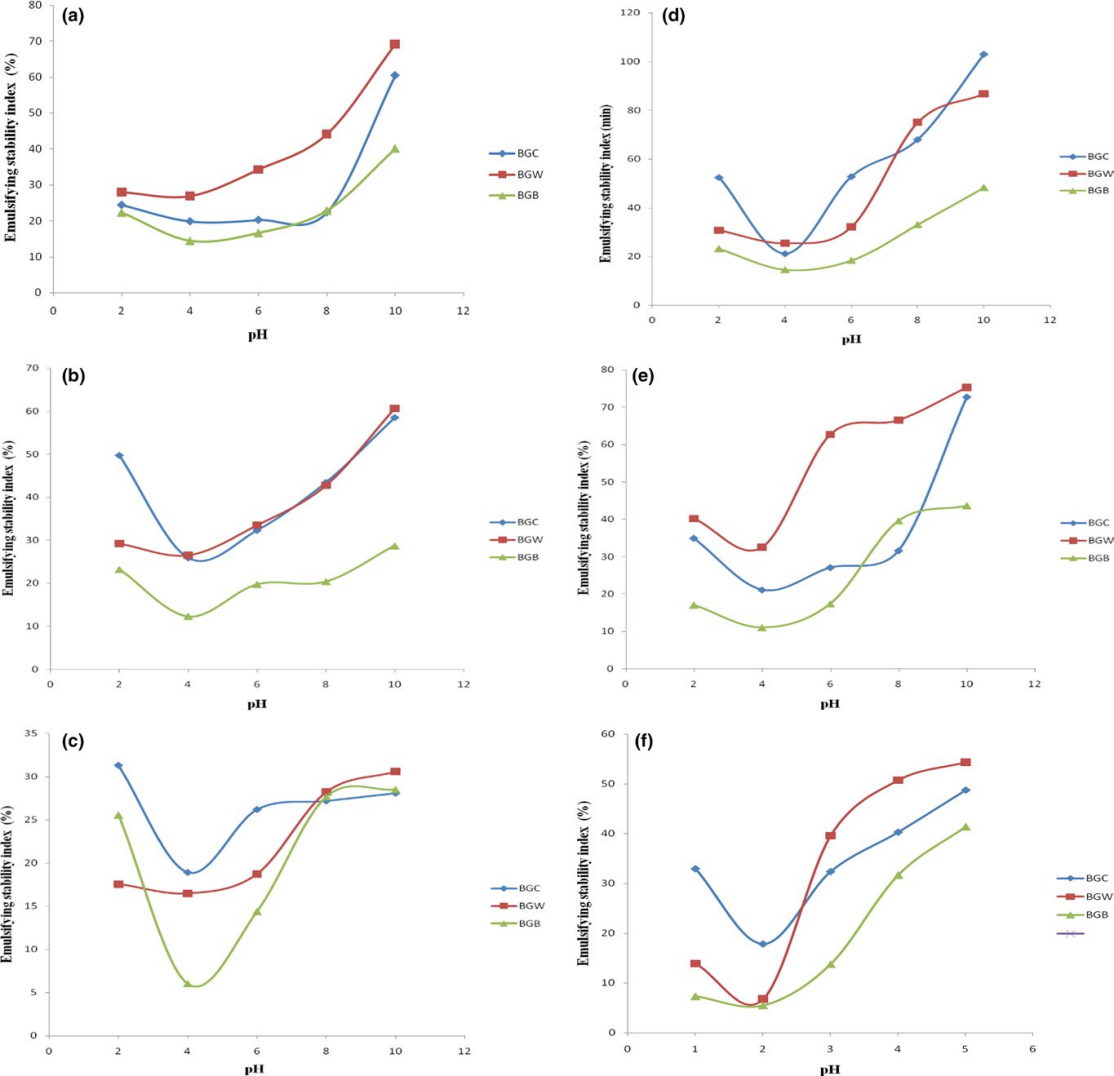
Emulsifying stability index (%) of defatted and protein concentrate of Bambara groundnut cultivars at (a, d) 0.0 mol/L, (b, e) 0.5 mol/L, and (c, f) 1.0 mol/L NaCl concentration as a function of pH

Emulsifying activity index (EAI) and emulsifying stability index (ESI) of defatted Bambara groundnut and protein concentrate flour was measured as a function of pH and NaCl concentration. Various concentrations of sodium chloride salt affected EAI and ESI of Bambara groundnut flour samples as shown in Figure 2 and 3, respectively. The lowest EAI of defatted BGC, BGW, and BGB at NaCl concentrations of 0.0, 0.5, and 1.0 mol/L were 2.80, 3.32, and 4.70 m^2^/g and it occurred around their isoelectric region (about pH 4.0). EAI of defatted BGC, BGW, and BGB increased at pH values above this region. The highest EAI of defatted BGC, BGW, and BGB at 0, 0.5, and 1.0 mol/L NaCl concentrations were 34.43, 38.06, and 32.76 m^2^/g and occurred at the alkaline pH of 10.0. Aremu, Olonisakin, Bako, and Madu (2008) reported that emulsion activity depends mostly on salt concentration and the type of the salt under consideration. The lowest EAI of protein concentrate BGC, BGW, and BGB at different concentrations of 0.0, 0.5, and 1.0 mol/L were 3.39, 3.59, and 3.26 m^2^/g, respectively, at their isoelectric pH of 4.0 with concomitant increase in emulsion activity below and above this pH. The highest EAI of protein concentrate BGC, BGW and BGB at 0.0, 0.5 and 1.0 mol/L were 40.40, 45.47 and 34.08 m^2^/g, respectively, at pH 10. However, the highest emulsion stability index (ESI) of defatted BGC, BGW, and BGB at 0.0, 0.5, and 1.0 mol/L were 60.52, 69.16, and 40.08%, respectively, at pH 10.0. The lowest ESI of defatted BGC, BGW, and BGB were 18.91, 16.47 and 6.04%, respectively, at their isoelectric region of pH 4.0. There was an increase in the ESI values above and below this region. Meanwhile, the lowest ESI of Bambara groundnut concentrate of samples BGC, BGW, and BGB were 17.88, 6.73, and 5.52%, respectively while the highest ESI of samples BGC, BGW, and BGB were 103.04, 86.78, and 48.36%, respectively. Good emulsifying activity of a protein is related to high solubility (El Nasri & El Tinay, 2007) while the pH‐emulsifying properties profile resembles the pH‐solubility profile (Ogunwolu, Henshaw, Mock, Santros, & Awonorin, 2009). This is because at isoelectric pH, most food proteins are sparingly soluble; poorly hydrated, lack electrostatic repulsive forces and are generally poor emulsifier. At this pH, the net charge of peptide will be minimized and peptide movement to the interface would not be rapid since the lowest solubility occurred at the isoelectric point. In the formation of stable emulsion, (Aremu et al., 2008) reported three mechanisms that may appear to be involved: (1) reduction of interfacial tension; (2) formation of a rigid interfacial film; and (3) electrical charge. The ability to lower the interfacial tension between water and oil in the emulsion is based on the surfactancy of proteins (Oshodi & Ojokan, 1997). The ease with which protein can migrate to, adsorb at, unfold and rearrange at an interface is a function of the surface activity and presumably salts reduce the surface activity of the flour and thereby increase the interfacial tension which leads to decrease in emulsion capacity. Charge repulsion between the proteins may also be reduced by salt which will in‐turn enhance hydrophilic association at the interface (Kinsella, Damodaran, & German, 1985). At isoelectric region, the decrease in emulsion stability may be due to increase contact leading to coalescence which thereby reduces stability (Parker, 1987). When moved away from their isoelectric pH, these proteins may, however, be effective emulsifiers.

### 3.5 Effect of pH and NaCl concentration on foam capacity and stability

**Figure 4:**
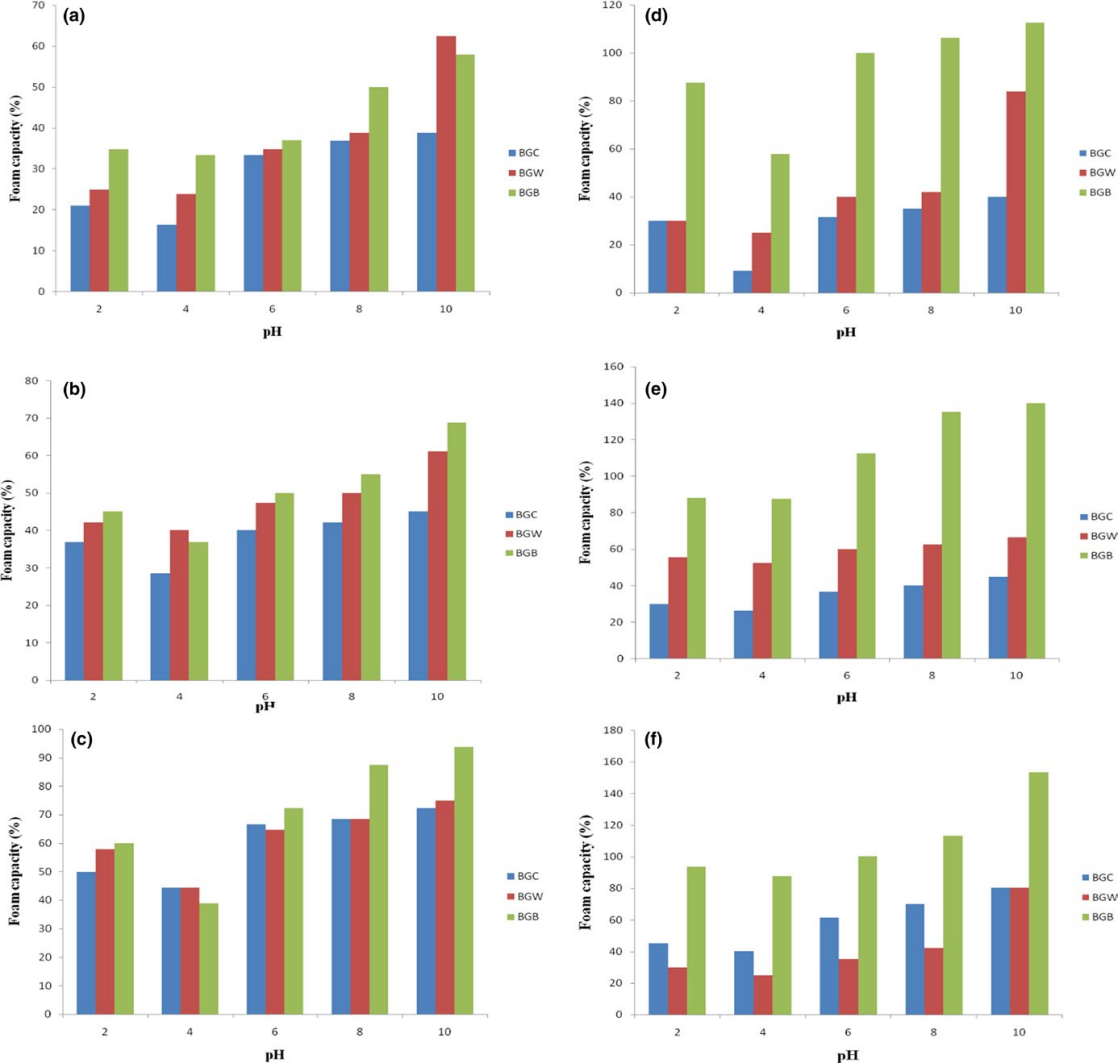
Foaming capacity (%) of defatted and protein concentrate of Bambara groundnut cultivars at (a, d) 0.0 mol/L, (b, e) 0.5 mol/L, and (c, f) 1.0 mol/L NaCl concentration as a function of pH

**Figure 5:**
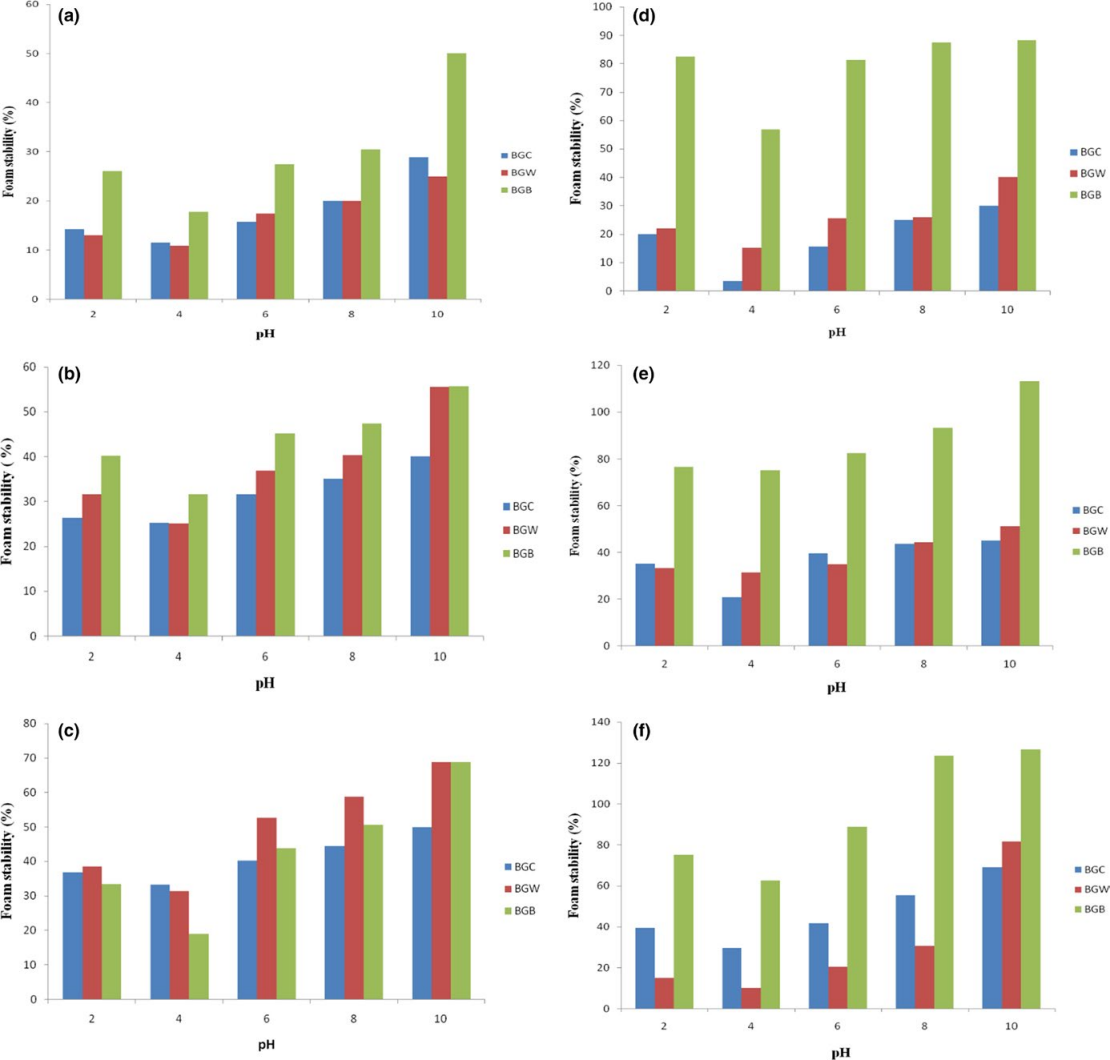
Foaming stability (%) of defatted and protein concentrate of Bambara groundnut cultivars at (a, d) 0.0 mol/L, (b, e) 0.5 mol/L, and (c, f) 1.0 mol/L NaCl concentration as a function of pH

The results showing the effect of pH and salt concentration on the foam capacity (FC) and foam stability (FS) of defatted and protein concentrate flour of samples BGC, BGW, and BGB are depicted in Figure 4 and 5, respectively**.** Figure 4a shows that the maximum FC at 0.0 mol/L sodium chloride concentration occurred at pH 10 for defatted Bambara groundnut flour samples BGC, BGW, and BGB and the values were 38.89, 62.5 and 57.89%, respectively. The lowest FC for samples BGC, BGW, and BGB were 16.32, 23.91, and 33.33%, respectively, and it occurred at pH 4.0. The FC of Bambara protein concentrate samples of BGC, BGW, and BGB at pH 10 and 0.0 mol/L NaCl concentration were 40.00, 84.00, and 112.50%, respectively, while it was 9.08, 25.00, and 57.89%, respectively, at pH 4.0 (Figure 4d). As the NaCl concentration increased from 0.0 to 1.0 mol/L, the FC of the samples also increases at all pH values. Generally, the defatted Bambara groundnut samples of BGC, BGB, and BGW exhibited a similar pattern of foaming capacity at the different salt concentrations and pH values to those of Bambara protein concentrates samples (BGC, BGW, and BGB). The differences in foaming properties of defatted BGC, BGB, and BGW and their concentrate samples may be due to the level of proteins present in their flour. The increase in solubility and surface activity of the soluble protein after removing lipids may be due to the improvement in foaming properties at higher pH (Jitngarmkusol, Hongsuwankul, & Tananuwong, 2008) while at aqueous phase, greater concentration of soluble protein could enhance foam formulation (Eltayeb et al., 2011). The increase in foaming properties observed at alkaline pH of walnut protein isolate and concentrate (Mao & Hua, 2012) and kariya protein hydrolysates Adiamo, Gbadamosi, and Abiose (2016b) in the report of, agrees to the assertion that foaming properties are pH dependent. The two new varieties of Bambara groundnut samples (BGB and BGW) showed superior foaming capacities over the commercial sample.

The FS of BGC (45.00%), BGW (66.66%), and BGB (140.00%) at pH 10 and 0.05 mol/L salt concentration were lower than the values of 80.25, 80.27, and 153.33%, respectively obtained at 1.0 mol/L salt concentration (Figure 5). The FS of defatted samples BGC (28.89%), BGW (25.00%), and BGB (50.03%) increased to 40.00, 55.56, and 55.58%, respectively at pH 10 and 0.5 mol/L NaCl concentration while it was also increased to 50.00, 68.73, and 68.75%, respectively, at 1.0 mol/L NaCl concentration. The FS of protein concentrate BGC, BGW, and BGB were 68.89, 81.58, and 126.67%, respectively, at pH 10 and 1.0 mol/L salt concentration while it was lower at pH 10 and 0.0 mol/L salt (30.00, 40.22, and 88.24%). Generally, the FS followed a similar pattern to foaming capacity where the maximum and minimum FS occurred at pH 10. Also sample BGB of both the defatted and concentrates exhibited the highest stability while the commercial sample (BGC) of the defatted and concentrates also exhibited the minimum FS. Increase in salt concentration from 0.5 mol/L to 1.0 mol/L also increased the FS including the FS around pH 4. The results obtained showed that the highest FS and FC was observed at alkaline pH 10 for sample BGB protein concentrate. A direct relationship was observed between foam capacity and foam stability (Mao & Hua, 2012). Large air bubbles surrounded by thinner and less flexible protein films could be formed by flour with high foaming ability while these air bubbles might be easier to collapse and consequently lowered the foaming stability (Jitngarmkusol et al., 2008). The results obtained in this study suggest that Bambara groundnut defatted flour and concentrate may be useful in food system to improve textural and leavening characteristics of foods such as ice cream, whipped topping, cakes and confectionery products where foaming properties are important as reported by Eltayeb et al. (2011).

## 4 CONCLUSION

In conclusion, the protein and fat contents of the new varieties of Bambara groundnut samples were found to be higher than the commercial sample. Also, the new varieties were found to contain higher levels of essential fatty acids, minerals and vitamins. Better functional properties were observed in the newly developed Bambara groundnut cultivars as compared to the commercial sample. The results obtained from this study have shown the potential for the industrial and household use of the newly developed Bambara groundnut cultivars into shelf stable protein products and could be a useful ingredient in food formulations.

## CONFLICT OF INTEREST

The authors declared no conflict of interest.
